# The Eighth Periodical International Ophthalmological Congress

**Published:** 1895-01

**Authors:** Alvin A. Hubbell

**Affiliations:** Buffalo, N. Y.


### THE EIGHTH PERIODICAL INTERNATIONAL OPHTHALMOLOGICAL CONGRESS, HELD AT EDINBURGH, AUGUST 7–11, 1894.

By ALVIN A. HUBBELL, M. D., Buffalo, N. Y.

(*Continued from page 299.*)

### OPENING OF THE CONGRESS.

The congress was opened Tuesday morning, August 7th, in the physiological department of Edinburgh University. On the nomination by Dr. Berry, of Edinburgh, Dr. Argyll-Robertson was elected president of the congress. On taking the chair he bade the members, on behalf of the committee, a hearty welcome to the ancient capital of Scotland. He pointed out how much the city owed to its beauty of situation and to its historical associations.

In particular, it was interesting to members as a seat of a university that had long been famous for the eminence of its medical school. Thirty-seven years had now elapsed since the first international ophthalmological congress, and they might pride themselves on being among the first to institute such meetings. Many changes had occurred in their ranks since the first meeting in 1857, and even since the last meeting at Heidelberg in 1888. They had to deplore the loss of two members who took the most prominent part in it—Prof. Donders and Prof. Becker, the former supreme in the domain of physiological optics, the latter the oculist to whom science was in particular indebted for his able investigations into the anatomy and pathology of the crystalline lens. He could not omit reference to one other misfortune which they had to deplore. At the last congress they rejoiced in one of the greatest, if not the greatest, physicist of the day, and one to whom ophthalmology was deeply indebted for the great progress it had made in recent times. Helmholtz was there, deeply interested in their work and giving them the advantage of his coöperation. Who was there that was present on that occasion that did not recall with affection and reverence the frank, simple and manly countenance of him to whom they owed the ophthalmoscope and whose genius had ranked him among the foremost of scientific men? Only the other day they were saddened with the news that their revered master was ill, stricken with paralysis. He was sure they all deeply sympathized with him in his affliction and prayed for a rapid recovery from his serious illness. The president congratulated those present upon the flourishing prospects of the congress. At the last meeting he said the propriety of continuing to hold an international ophthalmological congress was considered worthy of discussion by the president, Dr. Donders. The decision arrived at was that these meetings should not be discontinued, and it was to him a source of great satisfaction. This congress showed no signs of decline; it retained its full vigor and vitality. It was no weak, puny shoot of what might be viewed as a parent trunk, dependent upon it for its very existence; but it was a sturdy, vigorous, independent plant, full of the sap of vigor and life. They would prove traitors to their noble leaders, Von Graefe, Donders and Bowman, were they to allow the institution they were so deeply interested in to die. Might the international ophthalmological congress ever flourish, and might each successive meeting be more prosperous than the preceding one.

On motion of the president, Mr. Power, of London, and Mr. Swanzy, of Dublin, were elected vice-presidents of the congress; Dr. Geo. A. Berry, of Edinburgh, was elected secretary-general; Dr. Parent, of Paris, Dr. C. Hess, of Leipzig, and Dr. Fergus, of Glasgow, assistant secretaries. The following honorary presidents were elected: Prof. Panas, Dr. Meyer and Dr. Landolt, of Paris; Prof. Zehender, of Munich; Prof. Leber, of Heidelberg; Prof. Hansen-Grut, of Copenhagen; Prof. Reymond, of Turin; Prof. Snellen, of Utrecht; Dr. St. John Roosa and Prof. Knapp, of New York; Mr. Critchett, of London; Mr. Priestly Smith, of Birmingham; Dr. Little, of Manchester; Dr. Pridgin Teale, of Leeds, and Dr. Reid, of Glasgow.

The Lord Provost, Sir James Russell, was then introduced, and said it was a great pleasure to be present on that occasion and to extend to the congress a right hearty welcome to the ancient city of Edinburgh. He referred to Edinburgh as a favorite meeting-place for various bodies and a city largely interested in medicine. They had a university and medical school of which they were proud, a university which, unlike other Scottish universities, did not owe its origin to ecclesiastics or to the church. It was founded more than three hundred years ago by the king of Scotland and by the town council of the city, and the town council has ever since had the warmest interest in its prosperity. They were, therefore, probably more than any other town in the kingdom, deeply interested in the progress of medicine, and had a greater appreciation, and had a possibly greater knowledge of the men who were engaged in its practice, and they felt that in welcoming this congress they were welcoming those who ought to be and were their friends.

The president then said he was sure they had all listened with pleasure to the Lord Provost and appreciated his kindness in welcoming this congress. Perhaps they were not all aware that it was a very unusual circumstance in Edinburgh for a member of their own profession to hold the responsible office of the Lord Provost, and on that account, and also on account of the interest he had manifested, he asked the congress to confer upon his lordship an honor which was unusual and in their case unique, that was, to make him an honorary member of the Edinburgh congress. This was done.

The congress was then declared open for scientific work, and during the sitting of four days fifty communications were presented, as follows:

FIRST DAY, TUESDAY, AUGUST 7TH.

1. On the subconjunctival treatment of operative and traumatic wounds of the cornea, by Dr. SNELLEN, of Utrecht.

2. Remarks on cataract extraction, based on a recent series of 600 successive cases, by Dr. KNAPP, of New York.

3. Scleral puncture as an adjunct to iridectomy in the treatment of glaucoma, by Mr. PRIESTLY SMITH, of Birmingham.

4. Injuries of the eyes caused by pieces of copper or brass, by Dr. LEBER, Heidelberg.

5. Traumatic paralysis of the motor nerves, by Dr. PANAS, of Paris.

6. (*a.*) A new operation for ptosis. (*b.*) Treatment for the immediate cure of corneal ulcers, by DR. MULES, of Bowdon, Cheshire.

7. Drainage of the eye, by Dr. PFLÜGER, of Bern.

8. Method of relieving tension in chronic glaucoma, by Mr. WALKER, of Liverpool.

SECOND DAY, WEDNESDAY, AUGUST 8TH.

DEMONSTRATIONS.

9. Albuminuric retinitis, by Dr. DIMMER, of Vienna.

10. Contribution to the anatomy and physiology of the iris, by Mr. JULER, of London.

11. The histological changes which accompany the healing of perforating wounds of the sclerotic, by Dr. FRANKE, of Hamburg.

12. Orbital cyst with microphthalmus, by Dr. LAPERSONNE, of Lille.

13. Exhibition of aseptic collyria, aseptic case for dressings, and a new skiascope, by Dr. DARIER, of Paris.

14. Alteration in the cells of visual centers produced by exposure of the eyes to light, by Dr. GUSTAV MANN, of Edinburgh.

PAPERS.

15. Chorio-retinitis—clinical varieties, etiology, treatment, by Dr. ABADIE, of Paris.

16. Changes in the macula associated with retinal inflammation and edema, by Mr. GUNN, of London.

17. Recurrent, temporary, visual obscurations with ophthalmoscopic appearances observed during the obscurations, by Dr. BENSON, of Dublin.

18. Retro-choroidal hemorrhages after operations on the eye, by Dr. DUFOUR, of Lausanne.

19. Mechanism of accommodation, by Dr. TSCHERNING, of Paris.

20. Treatment of ocular diphtheria by crude petroleum, by Dr. VIAN, of Toulon.

21. Effect produced by the pressure of the lids upon the cornea, by Dr. BULL, of Paris.

22. Optic neuritis of tonsillary reflex origin, by Dr. MENACHO, of Barcelona.

THIRD DAY, THURSDAY, AUGUST 9TH.

DEMONSTRATIONS.

23. New method of hardening eye-preparations by formol, by Dr. LEBER, of Heidelberg.

24. Ulcers of the cornea produced by staphylococci, by Dr. BACH, of Würzberg.

25. Structure of the ganglion cells of the retina, by Dr. BACH, of Würzberg.

26. Nature of Schlemm's canal, by Dr. GUTMANN, of Berlin.

27. Two cases of serous cyst of the iris, by Dr. CLARK, of Columbus, Ohio.

PAPERS.

28. Operations for strabismus (strabotomy), by Dr. LANDOLT, of Paris.

29. Relative value of mercury and iodide of potassium in the treatment of ocular syphilis, by Dr. CHIBRET, of Clermont-Ferrand.

30. The best way of using mercury in ocular therapeutics, by Dr. DARIER, of Paris.

31. Correction of high degrees of myopia by removal of the crystalline lens, by Dr. THIER, of Aix-la-Chapelle.

32. Indications for removal of the crystalline lens in high myopia, by Dr. FUKALA, of Pilsen.

33. Operation for occlusion of the pupil in aphakia, by Dr. NOYES, of New York.

34. Anatomy of the ciliary ganglion, by Dr. MICHEL, of Würzberg.

35. New metric or dioptral system of measuring and designating prisms, by Dr. BURNETT, of Washington.

36. Treatment of certain forms of optic-nerve atrophy by antipyrin, by Dr. VALUDE, of Paris.

FOURTH DAY, FRIDAY, AUGUST 10TH.

DEMONSTRATIONS.

37. Illustrations of leprous diseases of the eye, by Dr. BORTHEN, of Drontheim.

38. Apparatus for skiascopy, by Dr. RISLEY, of Philadelphia.

39. Granulation and chalazion forceps, by Dr. AYRES, of Cincinnati.

40. Case of lymphoma of the four eyelids cured by internal use of arsenic, by Mr. BRONNER, of Bradford.

PAPERS.

41. Maturation of senile cataract by preliminary iridectomy with trituration of the lens, by Mr. MCHARDY, of London.

42. Sympathetic ophthalmia after sarcoma of the choroid, by Dr. NIEDEN, of Bochum.

43. Radical cure of strictures of the nasal duct, by Dr. THEOBOLD, of Baltimore.

44. Treatment of congenital hydrophthalmia, by Dr. STŒLTING, of Hanover.

45. Treatment of conjunctival granulations by electrolysis, by Dr. MALGAT, of Nice.

46. Associated paralysis of movements of the eyes upwards, downwards and for convergence, by Dr. SAUVINEAU, of Paris.

47. Operative cure of persistent tabetic ophthalmoplegia, by Dr, CHEVALLEREAU, of Paris.

48. Oblique astigmatism and the oblique muscles, by Dr. SAVAGE, of Nashville, Tenn.

49. A study of abnormalities of ocular balance, by Dr. RISLEY, of Philadelphia.

50. The relation of the function of accommodation to that of convergence, by Dr. STEVENS, of New York.

For purposes of abstract and analysis these fifty communications and the discussions which they elicited, may be grouped into those which treated of histological anatomy and physiology, pathological anatomy, etiology, particular diseases, injuries and accidents, medical therapeutics, operative therapeutics, instruments, miscellaneous.

### HISTOLOGICAL ANATOMY AND PHYSIOLOGY.

JULER (10) exhibited microscopical preparations verifying the works of Schwalbe, Treacher Collins, and others regarding the existence of a dilatator pupillæ muscle. These dilator fibers extend in the posterior layer of the iris from the ligamentum pectinatum, without to the sphincter of the pupil within. This muscle has two actions: (1) it dilates the pupil; (2) by its action on the ligamentum pectinatum it tends to close the spaces of Fontana. Recently its presence has been established physiologically by the experiments of Langley and Anderson.

MANN (14) gave an exceedingly interesting demonstration purporting to show the physiological changes which take place in the cells of the visual centers of the brain through the action of light on the retina. It had been shown that the nuclei of the sympathetic nerve-cells enlarge as the result of stimulation for fifteen minutes and become shrivelled after prolonged stimulation. He had made numerous preparations showing that in all cases the nuclei first enlarge, and that both cells and nuclei lose their chromatin, in part, on stimulation of fifteen minutes to five hours, but if continued nine hours the nuclei shrivel. The same holds good for motor cells of the brain and spinal cord. Excitation of the retina by light produces the changes referred to in the cells of the optic apparatus, especially in the ganglion cells of the rods of the retina, in the cells of the external geniculate bodies, in those of the anterior corpora quadrigemina, and the cells of Ramon y Cajal between the molecular and the small pyramidal layer of the lower aspect of the occipital lobes in rabbits.

Bach (25) described the minute structure of the retina and held that its ganglion cells do not have a fascicular disposition, but that they are granular and their contents nuclear.

Gutmann (26) had injected colored liquids into the canal of Schlemm in 35 cadavers, with the result of absolutely confirming the opinion of Schwalbe that a communication exists between this canal and the anterior chamber of the aqueous humor.

Michel (34) showed some excellent microscopical preparations of the ciliary ganglion, demonstrating that in its minute anatomy it resembles a sympathetic ganglion. Its cells, like those of the sympathetic, are multipolar, with axis-cylinder and other numerous prolongations. Each nerve-cell is surrounded by a fibrous investment, which is regarded as the termination of the fibers of the oculo-motor nerve. Thus the pupillary and ciliary muscles are indirectly acted upon by the oculo-motor.

Stevens (50), after a thorough investigation of the relation between accommodation and convergence, has come to conclusions quite at variance with those generally accepted. He holds (1) that there is no essential relation between the two functions, the connection existing incidentally and as the result of habitual association. (2) The proportion of cases of convergent strabismus associated with hypermetropia, and divergent strabismus associated with myopia, has been exaggerated. (3) The cases of strabismus which are relieved by convex or concave spherical glasses are cases of hypertropia or hyperphoria, and the relief obtained is largely due to their action as vertical prisms. (4) His observations have led him to believe that excessive accommodation does not directly cause convergent strabismus.

Tscherning (19), in speaking of the mechanism of accommodation, said that his experiments had demonstrated that the increase of refraction of the eye during accommodation diminishes toward the periphery. At a distance from the axis of 2.5 millimeters the accommodation is only one-half of that at the center. This is due to the fact that the anterior surface of the lens, during accommodation, becomes pointed. The same change of the lens may be produced in animals by traction on the ciliary zone.

Chibret confirmed the theory of Tscherning.

### PATHOLOGICAL ANATOMY.

Dimmer (9) called attention to the fact that in albuminuric retinitis the degenerative changes were greatest in extent and intensity in the macular region, because the conditions are here the most unfavorable for the nutrition of the retina.

Franke (11) gave a most interesting and instructive demonstration, microscopically, of the mechanism by which perforating wounds of the sclera heal.

Lapersonne (12) exhibited microscopic preparations of an orbital cyst with a microphthalmic eyeball, the walls of which were a well-developed but degenerated retina.

### ETIOLOGY.

Bull (21) regarded pressure of the lids upon the cornea as an important factor in causing certain forms of ocular fatigue, which he calls tarsal asthenopia, and which may be experienced by many persons on reading in bed.

Savage opposed this explanation of the asthenopia caused by reading in the recumbent posture, and referred it to the excessive work which such a position brings upon the inferior recti and superior obliques.

Burnett had found, some years ago, that pressure of the lids caused an astigmatism which he was not able to correct by any cylindrical lens, and that the changes by lid-pressure produced marked changes of the cornea-curve, as shown by the ophthalmometer.

Power cited a case in which, after a blepharoplasty, the new lid so caused increased pressure on the cornea as to produce a high degree of astigmatism.

Menacho (22) reported a case of double optic neuritis occurring in a young girl two weeks after the onset of catarrhal tonsilitis. She was totally blind and remained so for two days, when the sight promptly returned after the excision of both tonsils, and within a month she saw as well as ever. He looked upon the case as reflex in character, the reflex action passing through the glosso-pharyngeal and sympathetic nerves by way of the pharyngeal plexus.

Borthen (37) exhibited a series of photographs illustrating the effects of leprosy upon the eyes.

Nieden (42) reported a case of sympathetic ophthalmia following choroidal sarcoma. The sarcoma contained cocci, both singly and in groups.

Deutschmann, in the discussion, said he had made a microscopical examination of the eyeball and optic nerve, and had found microörganisms both in the immediate neighborhood of the tumor and in the sheath of the optic nerve. He thought this would sustain the diagnosis of a "migratory" inflammation.

DISEASES.

Abadie (15) described chorio-retinitis in a clear and systematic manner. He presented very little that was new. He emphasized the disproportion which often exists between the apparent lesions as seen by the ophthalmoscope and the functional disturbances of vision, the former being prominent while the vision is nearly unaffected in some, and in others the reverse. He considered the best treatment to be hypodermic injections of bichloride of mercury and peptonate or cyanide of mercury. In rebellious forms he had found it advantageous to combine with it subconjunctival injections of one or two drops of 1 to 1000 solution of bichloride mercury. He protested earnestly against the indiscriminate use of iodide of potassium.

Deutschmann made some strictures on the paper and favored subconjunctival injections in syphilitic cases. He had also obtained good results from iodide of potassium.

Panas, while not sharing the author's opinion as to the absolute inefficiency and disastrous results of iodide of potassium, agreed with him perfectly as to the superiority of the mercurial treatment.

Gunn (16) called attention to various lines and folds which radiate from the macula in cases of edema of the retina, whether from chorio-retinitis, anemia, retinal thrombosis or other conditions. These folds often remain after the disappearance of the edema and inflammation.

Thompson, of Indianapolis, had seen many cases of the kind, but they were found, in the vast majority of cases, in young women from 19 to 35 years of age.

Benson (17) reported a case of recurrent, temporary, visual obscuration in a seaman, who was a great smoker and who had a history of malaria and acute rheumatism. He had had these attacks for four years, sometimes the obscuration being total and sometimes in a single section of the visual field, and they would last from two to five minutes. The left eye was the one generally affected. During an obscuration, the ophthalmoscope showed "total bloodlessness of part of the retina, the anemic part changing in position." He believed the attacks to be due to spasm of one or more branches of the central retinal artery.

Noyes added a case of total blindness which lasted twenty-four hours, and in which the retinal arteries were wholly empty and the nerve extremely pale. Inhalations of nitrite of amyl relieved the condition.

Thompson thought that ophthalmic migraine might be but one remove from the condition described.

Clark (27) described two cases of cyst of the iris, one of which was congenital and the other developed without traumatism.

Bronner (40) reported a case of lymphomata of all four eyelids in a man 52 years of age. He saw him first in 1885, when there was found an elastic, elongated swelling of the left lower lid over which the skin was normal and freely movable. This tumor was removed and did not recur in three years. Then the four lids were found to be enlarged, and there was also a tumor of the right hard palate and the right submaxillary gland. Iodide of potassium and mercurial inunctions were ordered, but the tumors increased in size. In 1890 and 1891, Dr. Teale removed those in both lower lids. In 1891, fifteen minims of liquor arsenicalis and five minims of tincture of opium were given three times a day and in two or three weeks all the tumors had diminished in size. As soon as the arsenic was discontinued, the tumors began to increase, but disappeared again as soon as the medicine was resumed. Microscopic preparations showed the growths to be true lymphoma.

Sauvineau (46) handed in a paper to the congress describing a clinical type of ocular paralysis, in which there exists a paralysis of the movements of the eyes up and down and of convergence, while the movements from side to side are normally preserved. This form of paralysis, which was described by Parinaud in 1883, may begin suddenly and persist indefinitely without change. The lesions are probably seated in the supra-nuclear centers of coördination (subependymal grey substance of the quadrigeminal bodies). He had proved in 1892 that a lesion here produces paralysis of different associated and conjugate movements of the eyes, constituting a variety of ophthalmoplegia—supra-nuclear ophthalmoplegia. These paralyses are, therefore, always binocular. The diplopia, which is frequently present, and which when it exists offers nothing characteristic, has only a secondary importance in these forms of central paralysis.

Risley (48) read a most interesting paper giving the results of extended observation and study of ocular inequilibrium. He said there were two groups of anomalies of ocular equilibrium: Congenital anomalies of the eyes, and anomalies of the muscular apparatus. He desired to call attention only to the group in which simple vision is maintained by a constant tension of the eye which suffers from the anomaly. In most cases the correction requisite for the relative insufficiency should be effected gradually and progressively and in such a manner that the patient in the meantime learns to use his convergence. In absolute insufficiency the only remedy is tenotomy or muscular advancement.

(The next and final installment of this report will embody the most interesting part of the proceedings of the congress—namely, subjects pertaining to ocular therapeutics, both medical and surgical.—A. A. H.)

*Errata.*—Page 299 (Dec. No.), line eleventh from top, for 1880 read 1888.

